# Stability Compensation Design and Analysis of a Piezoelectric Ceramic Driver with an Emitter Follower Stage

**DOI:** 10.3390/mi14050914

**Published:** 2023-04-23

**Authors:** Xueliang Wang, Nan Zheng, Fenglong Wei, Yue Zhou, Huaijiang Yang

**Affiliations:** 1 Changchun Institute of Optics, Fine Mechanics and Physics, Chinese Academy of Sciences, Changchun 130033, China; 2 University of Chinese Academy of Sciences, Beijing 100049, China; 3 Beijing Gopptix Technology Co., Ltd., Beijing 100176, China

**Keywords:** piezoelectric ceramic, stability compensation, driver, emitter follower

## Abstract

Piezoelectric ceramic has been widely applied in many fields because of its characteristics, and the performance of piezoelectric ceramic is determined strongly by its driver. In this study, an approach to analyzing the stability of a piezoelectric ceramic driver with an emitter follower stage was presented, and a compensation was proposed. First of all, using the method of modified nodal analysis and loop gain analysis, the transfer function for the feedback network was analytically deduced, and the cause of the instability of the driver was found to be the pole composed of the effective capacitance from the piezoelectric ceramic and the transconductance from the emitter follower. Then, a compensation involving a novel delta topology composed of an isolation resistor and a second feedback path was proposed, and its function principle was discussed. Simulations showed a correspondence between the analysis and the effectiveness of the compensation. Finally, an experiment was set up with two prototypes, one with compensation, and the other without compensation. Measurements showed the elimination of oscillation in the compensated driver.

## 1. Introduction

Piezoelectric ceramic is a transducer that transforms electrical energy into mechanical energy [1,2,3]. It has characteristics of high resolution, fast response, high stiffness, little magnetic interference, and little heat generation, making piezoelectric ceramic advantageous over conventional actuators in precise applications [4,5,6,7]. Piezoelectric ceramic has been widely applied in many fields, such as fine machining, semiconductors, life sciences, and so on [8,9,10,11].

A lot of research has been carried out about piezoelectric ceramic over the last few years. Ghenna et al. analyzed and designed a motor with piezoelectric ceramic for applications that require compact size, high blocking, and driving forces [12]. Wei et al. developed a three-degree-of-freedom actuator with piezoelectric ceramic to manipulate a large and heavy mirror in an optical system [13]. Massavie et al. utilized piezoelectric ceramic as a resonator to filter the second harmonic in power electronics, and effectively improved the efficiency of the filter [14]. The development of a novel hydraulic actuator, with its characteristic high bandwidth, was proposed by Li et al. [15]. Degefa et al. studied the mathematical model between the characteristics of piezoelectric sheets and piezoelectric stacks, and their geometric dimensions [16]. Zhou et al. and Yu et al. proposed modified Prandtl–Ishlinskii models to compensate for the asymmetric and rate-dependent hysteresis of piezoelectric ceramic [17,18]. Yeh et al. presented an output-feedback sliding-mode control scheme to suppress the unknown nonlinearity of piezoelectric ceramic to avoid complex hysteresis models [19]. Zhang et al. studied the rate-dependent hysteresis of piezoelectric ceramic, and designed a hybrid adaptive controller for a piezo-actuated stage for accurate positioning [20]. Roshandel et al. proposed a high-step-up, high-efficiency, high-power density DC-DC converter to drive a piezoelectric transmitter [21]. Pai et al. designed a driving circuit for piezoelectric ceramic by increasing the leakage inductance of the transformer core, which makes voltage gain changes insensitive to frequency changes [22]. Kobayashi and Kawakatsu presented a driving circuit for piezoelectric ceramic that has a comparatively larger surface area, with a high transient current of up to 100A [23]. Xu et al. developed a high-voltage operational amplifier based on the principles of general operational amplifiers, in order to realize a low-cost and high-performance driver for piezoelectric ceramic [24]. Drivers based on the principle of charge feedback control were separately proposed by Yang et al., Bazghaleh et al., and Jin et al., in order to improve the linearity of piezoelectric ceramic [25,26,27].

The above literature mainly focused on the application, modeling and control, and driver design of piezoelectric ceramic. Since piezoelectric ceramic is inherently capacitive, it tends to erode the driving system’s stability, which is the foundation of all the other performance features. The literature on this topic is relatively scarce, and this study is aimed at a systematic study of this problem. The manuscript is organized as follows: Section 2 first describes the materials of this research in detail and the preparation for subsequent analysis, after which an analysis for the driver is carried out, and the design of compensation is presented; Section 3 provides the simulation results and experimental results; in Section 4, the results and future research are discussed; finally, in Section 5 the conclusions are summarized for this study.

## 2. Materials and Methods

### 2.1. Piezoelectric Ceramic and Driver Circuit

In this research, the piezoelectric ceramic of P-887.51 from PI was driven as a demonstration, and its parameters are listed in Table 1.

**Figure 1:**
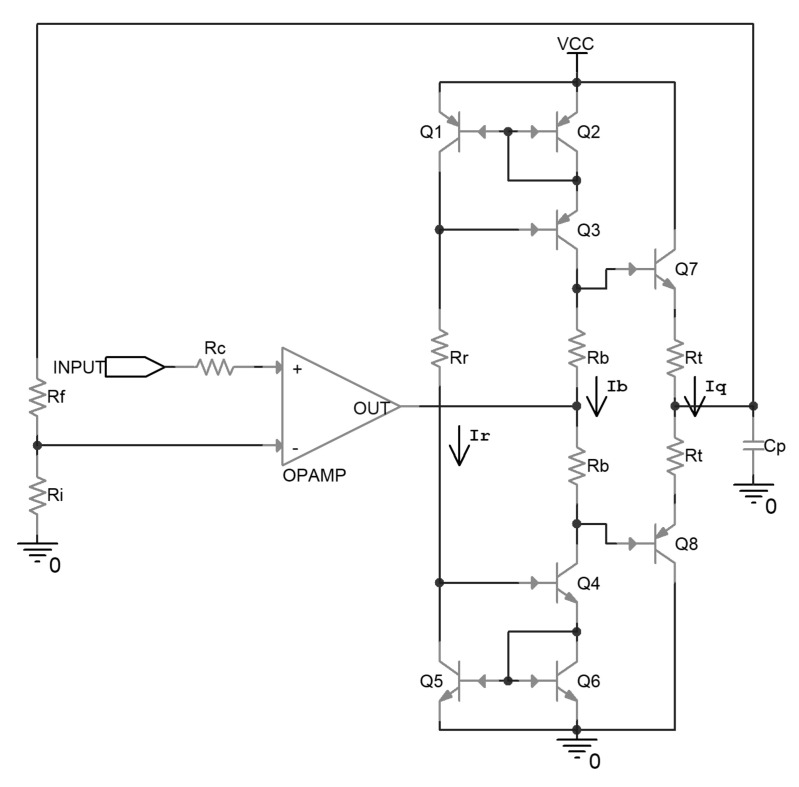
Schematic of an uncompensated piezoelectric ceramic driver.

The uncompensated driver circuit is shown in Figure 1. In this circuit, the operational amplifier is configured as a non-inverting amplifier, and amplifies the input signal to the desired voltage level according to the ratio between R_f_ and R_i_. On the non-inverting input, there is a series resistor R_c_, the value of which should be about R_f_ parallel with R_i_, to help to cancel out the adverse impact of the bias current of the operational amplifier input stage on the output accuracy.

Following the operational amplifier, there is a class-AB push-pull output stage composed of emitter followers, together with its biasing stage. The biasing stage is composed of Wilson current mirrors, which are implemented by Q1 to Q6, and set the bias current through R_r_. By the function of the current mirror, the bias current flows through R_b_; this generates a voltage difference between the base of the output transistors, and consequently determines the quiescent current flowing through the output transistors when no load is driven. The local negative feedback resistors R_t_ are implemented in series with the emitter of the output transistors, in case the output transistors break down because of the positive correlation between the collector current and the temperature. C_p_ is the effective capacitance of the driven piezoelectric ceramic.

The values of the components implemented in the above circuit are listed in Table 2.

### 2.2. Preparation for the Analysis

#### 2.2.1. Determination of the Parameters for the Transistor Model

It was assumed that a forward-biased transistor has a V_BE_ of about 0.65 V, and that the base of the output transistor and the output of the operational amplifier source or sink little current, so the bias current of the transistors involved in the driver can be calculated from Equation (1).

(1) $$\{\begin{array}{c} I_{r}=\frac{VCC-2V_{BE,Q1}-2V_{BE,Q4}}{R_{r}}\\ I_{b}=I_{r}\\ I_{b}R_{b}=I_{q}R_{t}+\frac{V_{T}}{2}\left(\ln\frac{I_{q}}{I_{S,Q7}}+\ln\frac{I_{q}}{I_{S,Q8}}\right) \end{array},$$

V_T_ denotes the thermal voltage, and I_s_ denotes the saturation current. Together with the above calculated bias current and parameters from the components’ datasheets and SPICE models, the hybrid-π models of the transistors have the following parameters, shown in Table 3.

#### 2.2.2. The Effective Impedance of the Current Mirror

Since the output impedance of the Wilson current mirrors connected to the base of the output transistors affects the AC analysis of the driver, it was necessary to have a check in advance. It can be found from Table 3 that the PNP transistor has a larger value of the parasitic capacitors, which is due to the fabrication process and material properties; thus, the top half current mirror composed of PNP transistors was taken as an example for analysis, and the bottom half was considered to have less effect.

**Figure 2:**
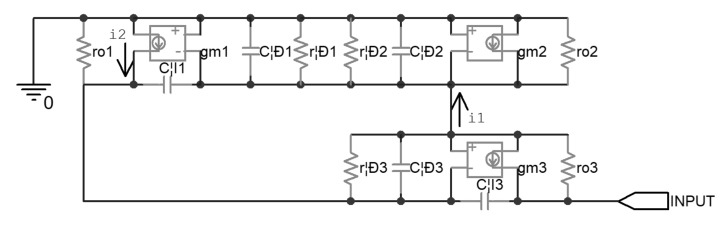
Equivalent circuit of the uncompensated piezoelectric ceramic driver.

Figure 2 shows the AC equivalent circuit of the above-mentioned current mirror. The transconductance of the diode-connected Q2 can be seen as a resistance with the value of 1/g_m2_, which is far less than r_π2_ and r_o2_; thus, i_1_ will mainly flow through this effective resistor, and the other resistors parallel to it can be omitted temporarily, in order to facilitate the analysis. Similarly, i_2_ was considered to mainly flow through r_π3_ and r_o1_, and were temporarily omitted. In this way the DC resistance R_0_ of the current mirror was found to be about 2.5 MΩ with Equation (2).

(2) $$R_{0}=\frac{1}{g_{m2}}*\frac{1}{2}+\frac{1}{2}*r_{\pi 3}*g_{m3}*r_{o3}\approx \frac{r_{\pi 3}*g_{m3}*r_{o3}}{2},$$

**Figure 3:**
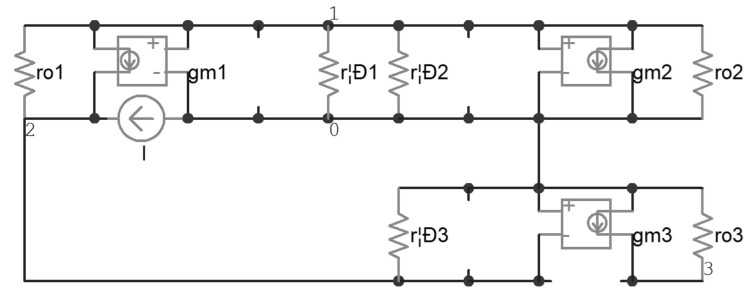
A demonstration for the calculation of R_μ1_.

Then, the zero-value time constant analysis [28] was carried out. The effective resistances seen by every single parasitic capacitor, when others are replaced by an open loop, are denoted by r_μ1_, r_π1_, r_π2_, r_π3_, and r_μ3_ respectively. The calculation of r_μ1_ is based on modified nodal analysis [29] and is demonstrated in Figure 3, in which the circuit can be described by Equation (3); r_μ1_ was found to be about 8.5 kΩ with Equation (4). In this way, all of the other effective resistances were calculated and, consequently, all of the time constants were calculated, as listed in Table 4. It was found that τ_μ3_ dominated, and the overall time constant τ was about 79 μs.

(3) $$\left[\begin{array}{ccc} \frac{1}{r_{\pi 1}}+g_{m1}+\frac{1}{r_{o1}}+\frac{1}{r_{\pi 2}}+g_{m2}+\frac{1}{r_{o2}} & -\frac{1}{r_{o1}} & 0\\ -\left(g_{m1}+\frac{1}{r_{o1}}\right) & \frac{1}{r_{o1}}+\frac{1}{r_{\pi 3}} & 0\\ 0 & g_{m3} & \frac{1}{r_{o3}} \end{array}\right]\left[\begin{array}{c} v_{1}\\ v_{2}\\ v_{3} \end{array}\right]=\left[\begin{array}{c} 0\\ I\\ 0 \end{array}\right],$$

(4) $$R_{\mu 1}=\frac{v_{2}}{I}=\frac{\left|\begin{array}{ccc} \frac{1}{r_{\pi 1}}+g_{m1}+\frac{1}{r_{o1}}+\frac{1}{r_{\pi 2}}+g_{m2}+\frac{1}{r_{o2}} & 0 & 0\\ -\left(g_{m1}+\frac{1}{r_{o1}}\right) & 1 & 0\\ 0 & 0 & \frac{1}{r_{o3}} \end{array}\right|}{\left|\begin{array}{ccc} \frac{1}{r_{\pi 1}}+g_{m1}+\frac{1}{r_{o1}}+\frac{1}{r_{\pi 2}}+g_{m2}+\frac{1}{r_{o2}} & -\frac{1}{r_{o1}} & 0\\ -\left(g_{m1}+\frac{1}{r_{o1}}\right) & \frac{1}{r_{o1}}+\frac{1}{r_{\pi 3}} & 0\\ 0 & g_{m3} & \frac{1}{r_{o3}} \end{array}\right|},$$

Thus, the effective impedance of the Wilson current mirror was modeled as a lumped capacitor C_0_ of 32 pF, in parallel with the DC resistance R_0_, which had a corresponding time constant of 79 μs.

### 2.3. Analysis of the Uncompensated Driver

**Figure 4:**
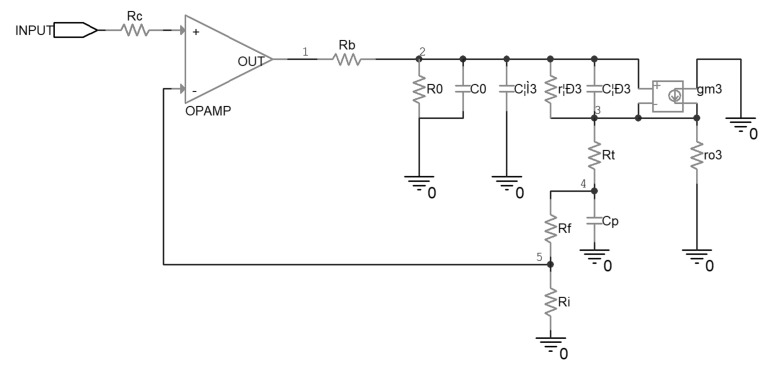
Equivalent circuit of the uncompensated piezoelectric ceramic driver.

Since in the output stage of the driver circuit either Q7 or Q8 will be conducting, depending on whether the output stage sinks from or sources to the piezoelectric ceramic, the equivalent circuit in these two cases will be symmetrical except for some slight difference and the top half will be analyzed. This approach led to the AC equivalent circuit of Figure 4, which turned out to be adequate for an approximate evaluation in the following analysis.

In this study, the tool of loop gain analysis [30] was used to evaluate the stability of the driver. The loop gain T is a product of the open-loop gain A_ol_ of the operational amplifier and the gain β of the feedback network. The A_ol_ of the operational amplifier, which has a DC magnitude of about 110 dB, with a dominating pole at about 2 Hz, was measured by the manufacturer, and can be found in the datasheet and the SPICE software model. Thus, the loop gain T is determined mainly by the feedback network gain β, and the following analysis and design in this research were based on the feedback network.

The sum of C_0_ and C_μ3_ was in the range of 100 pF, so they functioned only at considerably high frequency, and thus were ignored for an intuitive result. At moderately high frequencies, r_o3_ conducted little current from node 3 compared with the R_t_ path, and it was ignored in the analysis; for the same reason, R_0_ was ignored as well. The circuit of the feedback network can be described by Equation (5), and the transfer function of β can be calculated by Equations (6)–(8).

(5) $$\left[\begin{array}{ccc} \frac{1}{R_{b}}+\frac{1}{r_{\pi }}+sC_{\pi } & -\left(sC_{\pi }+\frac{1}{r_{\pi }}\right) & 0\\ -\left(sC_{\pi }+\frac{1}{r_{\pi }}+g_{m}\right) & sC_{\pi }+\frac{1}{r_{\pi }}+g_{m}+\frac{1}{R_{t}} & -\frac{1}{R_{t}}\\ 0 & -\frac{1}{R_{t}} & \frac{1}{R_{t}}+sC_{p} \end{array}\right]\left[\begin{array}{c} v_{2}\\ v_{3}\\ v_{4} \end{array}\right]=\left[\begin{array}{c} \frac{1}{R_{b}}v_{1}\\ 0\\ 0 \end{array}\right],$$

(6) $$\beta =\frac{v_{5}}{v_{1}}=\frac{R_{i}}{R_{i}+R_{f}}\frac{\left|\begin{array}{ccc} \frac{1}{R_{b}}+\frac{1}{r_{\pi }}+sC_{\pi } & -\left(sC_{\pi }+\frac{1}{r_{\pi }}\right) & \frac{1}{R_{b}}\\ -\left(sC_{\pi }+\frac{1}{r_{\pi }}+g_{m}\right) & sC_{\pi }+\frac{1}{r_{\pi }}+g_{m}+\frac{1}{R_{t}} & 0\\ 0 & -\frac{1}{R_{t}} & 0 \end{array}\right|}{\left|\begin{array}{ccc} \frac{1}{R_{b}}+\frac{1}{r_{\pi }}+sC_{\pi } & -\left(sC_{\pi }+\frac{1}{r_{\pi }}\right) & 0\\ -\left(sC_{\pi }+\frac{1}{r_{\pi }}+g_{m}\right) & sC_{\pi }+\frac{1}{r_{\pi }}+g_{m}+\frac{1}{R_{t}} & -\frac{1}{R_{t}}\\ 0 & -\frac{1}{R_{t}} & \frac{1}{R_{t}}+sC_{p} \end{array}\right|},$$

(7) $$\beta =\frac{R_{i}}{R_{i}+R_{f}}\frac{sC_{\pi }\frac{r_{\pi }}{1+r_{\pi }g_{m}}+1}{s^{2}C_{p}C_{\pi }\frac{r_{\pi }\left(R_{b}+R_{t}\right)}{1+r_{\pi }g_{m}}+sC_{p}\left(R_{t}+\frac{R_{b}+r_{\pi }}{1+r_{\pi }g_{m}}\right)+sC_{\pi }\frac{r_{\pi }}{1+r_{\pi }g_{m}}+1},$$

(8) $$\beta \approx \frac{R_{i}}{R_{i}+R_{f}}\frac{sC_{\pi }\frac{r_{\pi }}{1+r_{\pi }g_{m}}+1}{\left(sC_{\pi }\frac{r_{\pi }\left(R_{b}+R_{t}\right)}{R_{t}\left(1+r_{\pi }g_{m}\right)+R_{b}+r_{\pi }}+1\right)\left(sC_{p}\left(R_{t}+\frac{R_{b}+r_{\pi }}{1+r_{\pi }g_{m}}\right)+1\right)},$$

In this study, as Equation (8) shows, the two poles in β of the uncompensated driver were located at 890 Hz and 134 kHz, respectively, and the zero in β was located at 2.3 MHz. Since the first pole of β was at a rather low frequency, it lagged the phase of T rapidly, together with the dominating pole of A_ol_. This usually means a decrease in the phase margin of T and a poor transient response, or even oscillation, which will be verified in Section 3. This is the reason why compensation for the driver was necessary.

### 2.4. Design of the Compensated Driver

**Figure 5:**
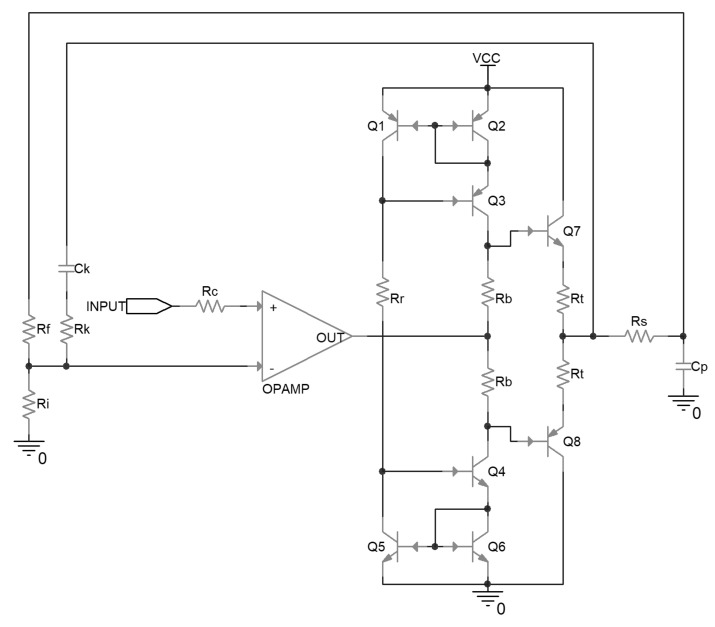
Schematic of the proposed compensated piezoelectric ceramic driver.

**Figure 6:**
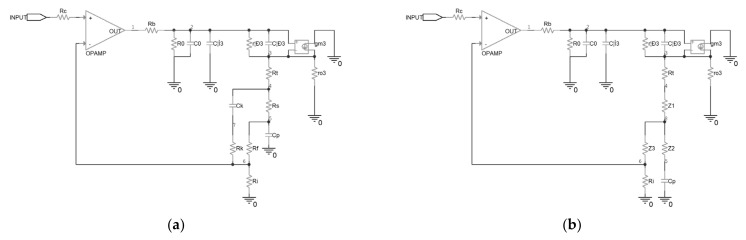
Equivalent circuit of the compensated piezoelectric ceramic driver: (**a**) before the delta-y transformation; (**b**) after the delta-y transformation.

To compensate for the stability of the original driver, a modified driver was proposed in this research, as shown in Figure 5. A series resistor R_s_ was inserted between the piezoelectric ceramic and the output of the driver, and a new feedback path was established by C_k_ and R_k_. In this case, the AC equivalent circuit of the top half driver is shown in Figure 6a. It can be seen that a delta topology is formed between node4, node5, and node6, because of the added components. A delta-y transformation was implemented to facilitate the analysis, and the schematic is shown in Figure 6b, in which the impedances, denoted as Z_1_, Z_2_ and Z_3_, were calculated with Equation (9).

(9) $$\left\{\begin{array}{c} z_{1}=\frac{R_{s}\left(1+sC_{k}R_{k}\right)}{1+sC_{k}\left(R_{s}+R_{f}+R_{k}\right)}\\ z_{2}=\frac{sC_{k}R_{s}R_{f}}{1+sC_{k}\left(R_{s}+R_{f}+R_{k}\right)}\\ z_{3}=\frac{R_{f}\left(1+sC_{k}R_{k}\right)}{1+sC_{k}\left(R_{s}+R_{f}+R_{k}\right)} \end{array}\right.,$$

The circuit of the feedback network can be described by Equation (10), and the transfer function of β can be calculated by Equations (11)–(13).

(10) $$\left[\begin{array}{ccc} \frac{1}{R_{b}}+\frac{1}{r_{\pi }}+sC_{\pi } & -\left(sC_{\pi }+\frac{1}{r_{\pi }}\right) & 0\\ -\left(sC_{\pi }+\frac{1}{r_{\pi }}+g_{m}\right) & sC_{\pi }+\frac{1}{r_{\pi }}+g_{m}+\frac{1}{R_{t}+Z_{1}} & -\frac{1}{R_{t}+Z_{1}}\\ 0 & -\frac{1}{R_{t}+Z_{1}} & \frac{1}{R_{t}+Z_{1}}+\frac{1}{Z_{2}+\frac{1}{sC_{p}}} \end{array}\right]\left[\begin{array}{c} v_{2}\\ v_{3}\\ v_{8} \end{array}\right]=\left[\begin{array}{c} \frac{1}{R_{b}}v_{1}\\ 0\\ 0 \end{array}\right],$$

(11) $$\beta =\frac{v_{6}}{v_{1}}=\frac{R_{i}}{R_{i}+Z_{3}}\frac{\left|\begin{array}{ccc} \frac{1}{R_{b}}+\frac{1}{r_{\pi }}+sC_{\pi } & -\left(sC_{\pi }+\frac{1}{r_{\pi }}\right) & \frac{1}{R_{b}}\\ -\left(sC_{\pi }+\frac{1}{r_{\pi }}+g_{m}\right) & sC_{\pi }+\frac{1}{r_{\pi }}+g_{m}+\frac{1}{R_{t}+Z_{1}} & 0\\ 0 & -\frac{1}{R_{t}+Z_{1}} & 0 \end{array}\right|}{\left|\begin{array}{ccc} \frac{1}{R_{b}}+\frac{1}{r_{\pi }}+sC_{\pi } & -\left(sC_{\pi }+\frac{1}{r_{\pi }}\right) & 0\\ -\left(sC_{\pi }+\frac{1}{r_{\pi }}+g_{m}\right) & sC_{\pi }+\frac{1}{r_{\pi }}+g_{m}+\frac{1}{R_{t}+Z_{1}} & -\frac{1}{R_{t}+Z_{1}}\\ 0 & -\frac{1}{R_{t}+Z_{1}} & \frac{1}{R_{t}+Z_{1}}+\frac{1}{Z_{2}+\frac{1}{sC_{p}}} \end{array}\right|},$$

(12) $$\begin{array}{c} \beta =\\ \frac{R_{i}}{R_{i}+R_{f}}\frac{1}{\left(sC_{k}\frac{R_{i}\left(R_{s}+R_{f}+R_{k}\right)+R_{f}R_{k}}{R_{i}+R_{f}}+1\right)}\frac{\left(sC_{\pi }\frac{r_{\pi }}{1+r_{\pi }g_{m}}+1\right)\left(s^{2}C_{k}C_{p}R_{s}R_{f}+sC_{k}\left(R_{s}+R_{f}+R_{k}\right)+1\right)}{\left(s^{2}C_{\pi }C_{p}\frac{r_{\pi }\left(R_{t}+R_{s}+R_{b}\right)}{1+r_{\pi }g_{m}}+sC_{p}\left(R_{t}+R_{s}+\frac{r_{\pi }+R_{b}}{1+r_{\pi }g_{m}}\right)+sC_{\pi }\frac{r_{\pi }}{1+r_{\pi }g_{m}}+1\right)}, \end{array}$$

(13) $$\beta \approx \frac{R_{i}}{R_{i}+R_{f}}\frac{\left(sC_{\pi }\frac{1}{g_{m}}+1\right)\left(sC_{p}\left(R_{s}||R_{f}\right)+1\right)\left(sC_{k}\left(R_{s}+R_{f}+R_{k}\right)+1\right)}{\left(sC_{\pi }\frac{r_{\pi }\left(R_{t}+R_{s}+R_{b}\right)}{\left(1+r_{\pi }g_{m}\right)\left(R_{t}+R_{s}\right)+r_{\pi }+R_{b}}+1\right)\left(sC_{k}\left(R_{i}||R_{f}+R_{k}\right)+1\right)\left(sC_{p}\left(R_{t}+R_{s}+\frac{r_{\pi }+R_{b}}{1+r_{\pi }g_{m}}\right)+1\right),},$$

Comparing the results from Equations (8) and (13), the following characteristics were found:

- The one original zero stemming from C_π_ stays unchanged and the two original poles stemming from C_p_ and C_π_ change slightly;
- The compensated circuit introduces two zeros stemming from C_p_ and C_k_ and one pole stemming from C_k_;
- The zeros and poles stemming from C_p_ and C_k_ makes the phase shift of β to be 0 degrees at high frequency under proper design;
- The first pole lagging the phase of β in the compensated driver stems from C_π_, and it is at a relatively high frequency. In contrast, the first pole lagging the phase of β in the uncompensated driver stems from C_p_, and it is at a relatively low frequency. Thus, the phase margin of the loop gain is increased.

In this research, R_s_ and R_k_ were designed to be 5.6 Ω and 10 kΩ, respectively, and C_k_ was designed to be 2 nF. As Equation (13) shows, the three poles in β of the compensated driver are located at 816 Hz, 3.9 kHz, and 122 kHz, respectively, and the three zeros in β are located at about 769 Hz, 9.3 kHz, and 2.3 MHz, respectively.

## 3. Results

### 3.1. Simulation Results

#### 3.1.1. Frequency Domain Simulation Results

**Figure 7:**
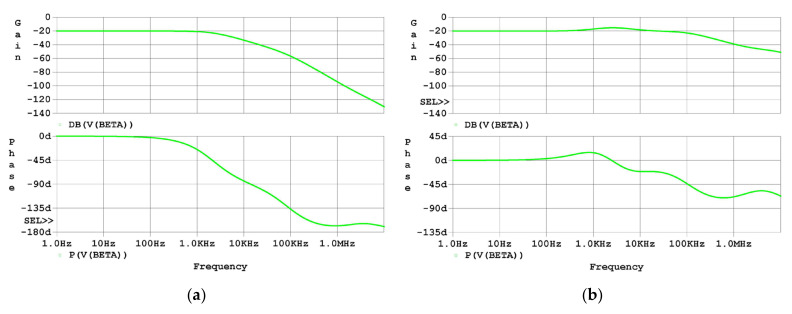
Frequency-domain simulation results of β: (**a**) Bode plot of β of the uncompensated driver; (**b**) Bode plot of β of the compensated driver.

The effectiveness of the analysis and design proposed in this study was first examined using SPICE software simulation in the frequency domain. The Bode plots of β of both the uncompensated driver and the compensated driver are shown in Figure 7. As the simulation results in Figure 7a graphically show, the first two poles in β are located at 2 kHz and 94 kHz, and the zero is located at about 2 MHz, which agrees approximately with the analysis in Section 2.3. As the simulation results in Figure 7b graphically show, the first three poles are located at about 1 kHz, 2 kHz, and 106 kHz, respectively, and the first three zeros are located at about 600 Hz, 12 kHz, and 2 MHz, respectively, which agree approximately with the analysis in Section 2.4.

The first pole lagging the phase of β of the uncompensated driver remarkably is at 2 kHz, whereas the first pole lagging the phase of β of the compensated driver remarkably is at 106 kHz, considering that a pole and a nearby zero cancel out with each other. As a result, the phase margin of the loop gain increased, and this can be seen from the graphic representation where the flat part of the gain is extended.

**Figure 8:**
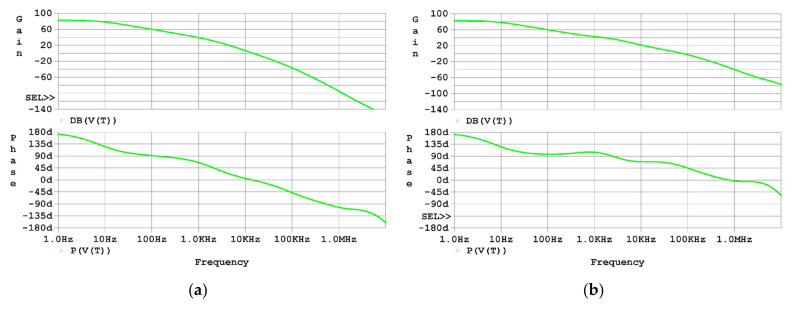
Frequency-domain simulation results of T: (**a**) Bode plot of T of the uncompensated driver; (**b**) Bode plot of T of the compensated driver.

Next, the Bode plots of T for both the uncompensated driver and the compensated driver are shown in Figure 8. As the simulation results in Figure 8a graphically show, there is no phase margin left for the loop gain of the uncompensated driver, which agrees with the analysis in Section 2.3. As the simulation results in Figure 8b graphically show, the phase margin for the loop gain of the compensated driver increased to about 50 degrees, which agrees with the analysis in Section 2.4.

#### 3.1.2. Time-Domain Simulation Results

**Figure 9:**
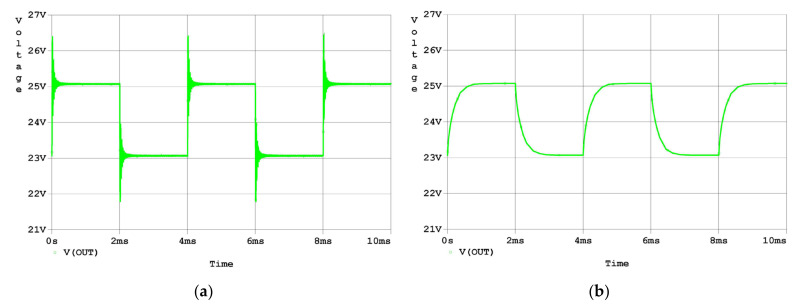
Time-domain simulation results of the response to the step input signal: (**a**) output voltage waveform of the uncompensated driver; (**b**) output voltage waveform of the compensated driver.

Finally, a transient simulation was carried out in the time domain to examine the effectiveness of the analysis and design proposed in this research. As the simulation results in Figure 9a graphically show, there is an overshoot of about 60% and a small oscillation in the response to a step input. As the simulation results in Figure 9b graphically show, the overshoot and oscillation in the response to a step input were eliminated by the compensation.

### 3.2. Experiment Results

#### 3.2.1. Setup of the Experiment

**Figure 10:**
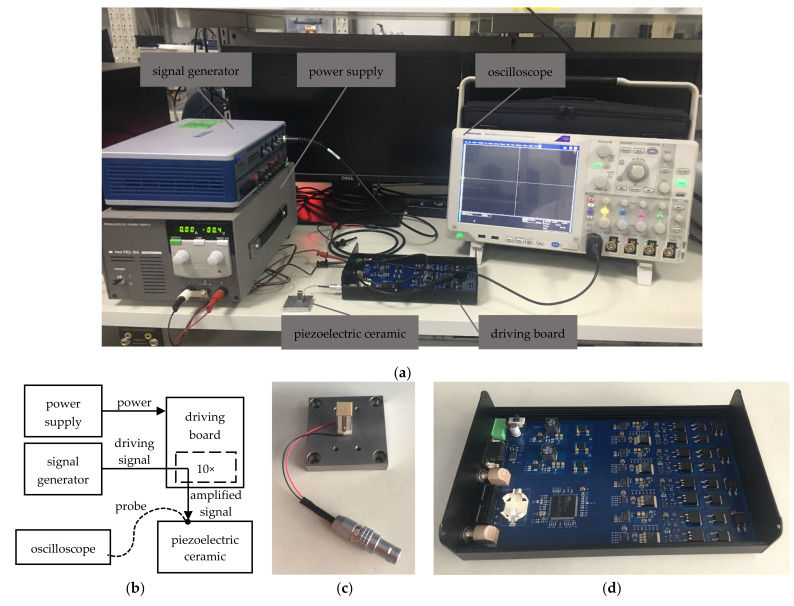
The experimental setup: (**a**) the instruments used in the experiment; (**b**) the schematic of the experimental setup; (**c**) the piezoelectric ceramic driven in the experiment; (**d**) the driving board under testing in the experiment.

To verify the effectiveness of the stability compensation proposed in this study, an experiment was set up as shown in Figure 10. A board was designed and manufactured with the compensated driver, and the uncompensated driver was modified on it. The board was powered by a PAN-A power supply from KIKUSUI. The VB-8054 from NI was used as a signal generator, and fed the testing signals for the driver. The piezoelectric ceramic PI-887.51 from PI was loaded to the driver with a cable, and the output voltage was probed and recorded by the oscilloscope DPO5034 from TEKTRONIX.

#### 3.2.2. Results of the Experiment

**Figure 11:**
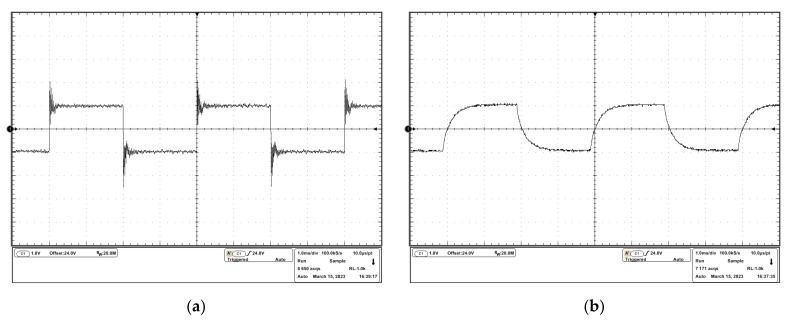
Experimental results of the response to the step input signal: (**a**) output voltage waveform of the uncompensated driver; (**b**) output voltage waveform of the compensated driver.

The experimental results are shown in Figure 11. The experimental results in Figure 11a show an overshoot of about 50% and a small oscillation in response to a step input signal. The experimental results in Figure 11b show a stable response to a step input signal because of the compensation, which agrees with the simulation results in Section 3.1.2.

**Figure 12:**
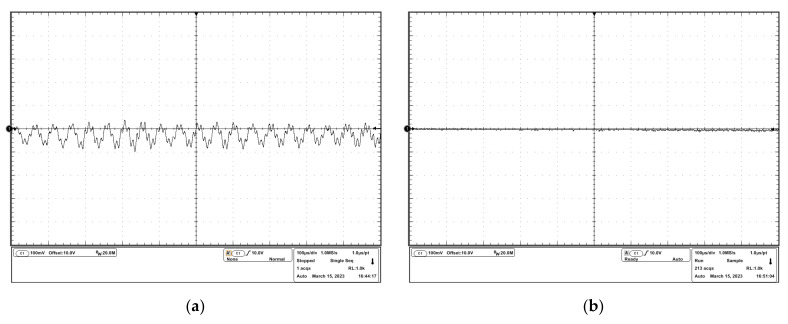
Experimental results of the response to the dc input signal: (**a**) output voltage waveform of the uncompensated driver; (**b**) output voltage waveform of the compensated driver.

**Figure 13:**
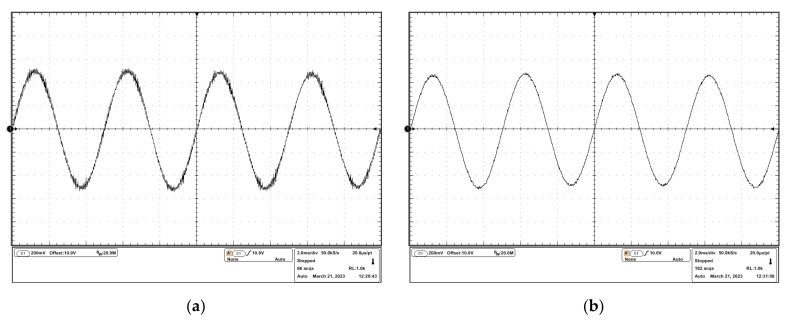
Experimental results of the response to the sinusoidal input signal: (**a**) output voltage waveform of the uncompensated driver; (**b**) output voltage waveform of the compensated driver.

To observe the oscillation more clearly, a DC input signal and a sinusoidal input signal were sourced into the drivers, and the experimental results are shown in Figure 12 and Figure 13. It can be seen that in the uncompensated driver, the oscillation resided on the desired driving waveforms. In the compensated driver, there were cleaner driving waveforms, which verified the effectiveness of the stability compensation proposed in this study.

## 4. Discussion

In this research, when carrying out the analysis, the influence of parasitic capacitors was ignored to obtain intuitive results. In fact, they not only determined the zeros and poles at high frequency, but also contributed fractionally to the zeros and poles at low frequency. This is why the analysis did not exactly correspond to the simulation.

From Equation (8), we can see that increasing the transconductance of the output transistor in the emitter follower stage shifted the pole stemming from the piezoelectric ceramic to a higher frequency, which helped to reduce the risk of oscillation. This was accomplished by increasing the quiescent current of the output transistor, which also meant more power dissipation, even when no load was driven, and consequently more heat. Thus, the designer should make a balance in a specific condition, and the analysis proposed in this research will help.

From the experimental data, we can see that the elimination of the oscillation is achieved at the cost of response speed. Hence, to design a driver for an application with the specific requirement of speed, a study of further optimization based on the compensation proposed in this research can be carried out in the future.

## 5. Conclusions

In this study, the stability problem of a piezoelectric ceramic driver with an emitter follower stage was analyzed, and a corresponding compensation was designed. The nature of the design was to optimize the structure of the transfer function of the loop gain by means of modifying the feedback network. The analytical expression of the feedback network was proposed so that the effect of every component was understood, and the consequence of changing them could be evaluated. Then, the analysis and the analytical expression were verified with the help of SPICE simulations, and the results showed their correspondence. Finally, an experiment was set up to verify the effectiveness of the compensation proposed in this study, and the results showed that the oscillation was eliminated by the compensation as designed.

## Figures and Tables

**Table 1:** Parameters of the piezoelectric ceramic of P-887.51.

Dimensions	Nominal Displacement	Blocking Force	Capacitance	Resonant Frequency
7 mm × 7 mm × 18 mm	15 μm	1750 N	3.1 μF	70 kHz

**Table 2:** The values of components implemented in the driver circuit.

Component	Value
OPAMP	OPA547
R_f_	102 kΩ
R_i_	11.3 kΩ
R_c_	10 kΩ
Q1 Q2 Q3	BC856
Q4 Q5 Q6	BC846
R_r_	90.9 kΩ
R_b_	1 kΩ
Q7	2SCR586J
Q8	2SAR586J
R_t_	1 Ω
C_p_	P-887.51

**Table 3:** The hybrid-π parameters for the transistors.

Component	r_π_	C_π_	g_m_	C_μ_	r_o_
BC856	13.0 kΩ	24 pF	19.2 mA/V	14 pF	27.4 kΩ
BC846	13.0 kΩ	8 pF	19.2 mA/V	4 pF	20.0 kΩ
2SCR586J	13.2 kΩ	1.3 nF	18.9 mA/V	188 pF	321 kΩ
2SAR586J	13.2 kΩ	1.3 nF	18.9 mA/V	337 pF	63 kΩ

**Table 4:** The time constants stemming from each capacitor.

τ_μ1_	τ_π1_	τ_π2_	τ_π3_	τ_μ3_
147 ns	744 ps	744 ps	252 ns	79 μs

## Data Availability

Not applicable.
